# Real-time analysis of liquid jet sample delivery stability for an X-ray free-electron laser using machine vision

**DOI:** 10.1107/S1600576724009853

**Published:** 2024-11-17

**Authors:** Jaydeep Patel, Adam Round, Raphael de Wijn, Mohammad Vakili, Gabriele Giovanetti, Diogo Filipe Monrroy Vilan e Melo, Juncheng E, Marcin Sikorski, Jayanth Koliyadu, Faisal H. M. Koua, Tokushi Sato, Adrian Mancuso, Andrew Peele, Brian Abbey

**Affiliations:** a School of Computing, Engineering and Mathematical Sciences La Trobe University Melbourne Victoria Australia; b European XFEL Schenefeld Germany; c Center for Free-Electron Laser Science CFEL Deutsches Elektronen-Synchrotron DESY Notkestrasse 85 22607Hamburg Germany; d Diamond Light Source Didcot United Kingdom; e Australian Synchrotron Australian Nuclear Science and Technology Organisation (ANSTO) Clayton Victoria Australia; f La Trobe Institute for Molecular Science (LIMS) La Trobe University Melbourne Victoria Australia; SLAC National Accelerator Laboratory, Menlo Park, USA

**Keywords:** liquid sample delivery, image processing, serial crystallography, machine vision algorithms, X-ray free-electron lasers

## Abstract

Automated evaluation of optical microscopy images of liquid jets, commonly used for sample delivery at X-ray free-electron lasers (XFELs), enables real-time tracking of the jet position and liquid jet hit rates, defined here as the proportion of XFEL pulses intersecting with the liquid jet. This method utilizes machine vision for preprocessing, feature extraction, segmentation and jet detection as well as tracking to extract key physical characteristics (such as the jet angle) from optical microscopy images captured during experiments. To determine the effectiveness of these tools in monitoring jet stability and enhancing sample delivery efficiency, we conducted XFEL experiments with various sample compositions (pure water, buffer and buffer with crystals), nozzle designs and jetting conditions. We integrated our real-time analysis algorithm into the Karabo control system at the European XFEL. The results indicate that the algorithm performs well in monitoring the jet angle and provides a quantitative characterization of liquid jet stability through optical image analysis conducted during experiments.

## Abstract 2

This paper describes real-time statistical analysis of liquid jet images for serial femtosecond crystallography (SFX) experiments at the European XFEL. This analysis forms one part of the automated jet realignment system for SFX experiments at the SPB/SFX instrument of the European XFEL.

## 1. Introduction

Serial femtosecond crystallography (SFX) (Barends *et al.*, 2022 ▸; Spence, 2017 ▸; Chapman *et al.*, 2011 ▸) has emerged as a critical new tool for determining macromolecular structures using small crystals at X-ray free-electron laser (XFEL) sources (Pellegrini, 2020 ▸; Decking *et al.*, 2020 ▸; Milne *et al.*, 2017 ▸; Kim & Yoon, 2009 ▸; Ko *et al.*, 2017 ▸). A major factor enabling the success of SFX experiments is the sample delivery. The most common approaches to sample delivery are fixed-target scanning, droplet on demand, high viscous extrusion injection (Berntsen *et al.*, 2019 ▸) and liquid jet injection (Weierstall, 2014 ▸; Cheng, 2020 ▸). In fixed-target scanning, crystals are mounted on a static substrate or chip that is then raster-scanned relative to the X-ray beam. This method can reduce sample consumption and is particularly useful for positioning larger crystals. Droplet-on-demand methods normally involve precise deposition of droplets containing crystals onto a substrate (often called a ‘tape drive’) or directly into the X-ray beam path using a droplet generator. This allows for controlled delivery of small volumes of sample (Henkel *et al.*, 2022 ▸). For liquid jet sample delivery, crystals are carried within a thin, gas-accelerated liquid stream of buffer solution to the XFEL beam (Weierstall, 2014 ▸). During each X-ray pulse, if a crystal is present within the interaction region of the beam and liquid jet, it diffracts the incident X-rays, which then typically results in the destruction of the crystal. To minimize the background signal, it is important that the liquid jet is relatively thin, ideally with a diameter comparable to the size of the crystals being measured. This can be achieved using various types of nozzle design, including the gas dynamic virtual nozzle (GDVN) and the double flow-focusing nozzle (DFFN) (Vakili *et al.*, 2022 ▸; Oberthuer *et al.*, 2017 ▸). Liquid jets can be used to deliver crystals of tens of micrometres in size down to nanocrystals with a diameter <100 nm. One of the advantages of the liquid jet system is that it is particularly beneficial for high-repetition-rate XFELs, such as the European XFEL (EuXFEL) which operates in the megahertz range, as it rapidly replenishes the sample at a rate comparable to the XFEL’s repetition rate. To achieve this, it jets the liquid stream at high velocities, often exceeding 50 m s^−1^ (Knoška *et al.*, 2020 ▸; Beyerlein *et al.*, 2015 ▸). One significant disadvantage of the liquid jet versus static delivery systems is that the position of the jet can drift over time due to instabilities in the jetting parameters (*e.g.* gas pressure), variations in the sample crystal size, or changes in the microchannels or nozzle [*e.g.* narrowing of the exit aperture due to accumulation of deposited material (Beyerlein *et al.*, 2015 ▸)]. If the movement of the jet becomes significant enough that it no longer intersects with the X-ray beam, this can lead to a critical loss of both beam time and sample (Patel *et al.*, 2022 ▸).

Movement in the position of the jet means that it is necessary to monitor the position of the jet to detect any systematic drift or large fluctuations in jet angle. The key parameters that determine spatial overlap with the XFEL beam are the jet angle and jet length. Spatial overlap is determined by scanning for gaps along the length of the liquid jet at the interaction point, confirmed by visual observation of jet explosions indicating beam interaction. The jet angle for alignment is then calculated using the trigonometric methods outlined by Patel *et al.* (2022 ▸). These parameters are interrelated and are critical for optimizing sample delivery in XFEL experiments. A change in jet angle impacts the degree of overlap between the X-ray beam and the liquid jet. If the jet length decreases, the interaction point will move closer to the breakup region – the area where the cohesive liquid stream disintegrates into droplets due to internal instabilities and external forces (see Fig. S1 of the supporting information). This, in turn, can impact the amount of time that overlap between the X-ray beam and liquid jet is maintained. Therefore, maintaining a constant jet angle and optimal jet length is essential to ensure the stability and reliability of the sample delivery system and thus maximize the hit rate. Often the user will employ optical microscopy images of the jet to evaluate these parameters during an experiment. However, continuous monitoring of the liquid jet for the entire duration of the experiment is both impractical and labour intensive. As a result, issues relating to jet instability are often only diagnosed after the jet has already moved out of alignment with respect to the X-ray beam.

To address this problem, we have developed an algorithm based on the principles of machine vision for continuously monitoring the physical characteristics of the jet, primarily focusing on changes in jet angle. The algorithm is also able to identify whether there is overlap (termed a ‘hit’) or no overlap (termed a ‘miss’) of the liquid jet and XFEL beam. Further details of the algorithm have been given by Patel *et al.* (2022 ▸). Here, we apply this algorithm to analyse a variety of XFEL data types to assess its effectiveness in identifying potential sources of jet instability that could increase the likelihood of the XFEL beam missing the liquid jet. The motivation behind this work is to be able to apply this algorithm to the real-time monitoring of liquid jets during actual experiments conducted at the EuXFEL. We aim to then use the output to inform an automated feedback loop for realigning the liquid jet with respect to the XFEL beam without requiring any manual intervention. In addition, the proposed algorithm provides an opportunity to automatically collect jetting statistics, which may be useful for further development and optimization of liquid jet sample delivery. The focus of the present work is to demonstrate a new tool for real-time monitoring of the liquid jet angle and its reliability across different combinations of jetting conditions, samples and nozzle types. This analysis could serve as a reference for future studies in this area.

## 2. Methods and materials

### 2.1. Algorithmic process

**Figure 1:**
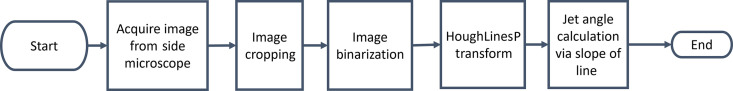
Flowchart of the algorithm for extracting jet angles from side microscope images.

The angle at which the liquid jet leaves the nozzle can be quantified via the analysis of images obtained from the optical side microscope. This microscope is used to view the liquid jet at the Single Particles, Clusters and Biology and Serial Femtosecond Crystallography (SPB/SFX) instrument at the EuXFEL (Mancuso *et al.*, 2019 ▸) and consists of a high-resolution microscope equipped with a high-speed sCMOS camera for image acquisition. This setup allows the capture of images and videos of the liquid jet in real time that provide critical insights into its behaviour and observation of the jet dynamics, crucial for experiments at the EuXFEL. Further details of the sample delivery setup and optical side microscope are given in Section 2.2. The image analysis was performed using a machine-vision algorithm that analysed the side microscope images frame by frame. Fig. 1 ▸ presents a flowchart summarizing each of the key steps from image acquisition to jet angle calculation for the machine-vision algorithm.

The first image acquisition step involves capturing frames from the side microscope camera. After the initial acquisition, the image is cropped to remove most of the background and the nozzle. The resulting cropped image contains only the section of the jet from the tip of the nozzle to the XFEL beam interaction point (Mancuso *et al.*, 2019 ▸; Beyerer *et al.*, 2016 ▸). Cropping the image to leave just this region of interest (ROI) helps to reduce the image processing time, while at the same time improving the signal-to-noise ratio around the interaction region (Kumar & Bhatia, 2014 ▸). After the ROI is extracted, the cropped image is binarized to further simplify the image analysis. This binarization is achieved via setting a threshold for the pixel intensity values for the image matrix. The thresholding value is set such that the darker pixels associated with the liquid jet are separated from the lighter background. This is carried out during the calibration step prior to the experiment. The threshold value is typically determined using a plotting tool available through the Karabo control system, the control system of the European XFEL, which is able to plot a histogram of the image pixel intensity values. Pixels with intensity above a set threshold are given the value ‘1’ and those below the set threshold are given the value ‘0’ (O’Gorman, 1994 ▸). Following image binarization, a Hough line transform is performed to detect any straight lines, at any orientation, within the image – typically these are expected to correspond to the liquid jet (Rasheed *et al.*, 2012 ▸). The OpenCV library (OpenCV Team, 2023 ▸) was used for this purpose and contains a function called ‘HoughLinesP’ which returns the coordinates of the lines detected in the image.

**Figure 2:**
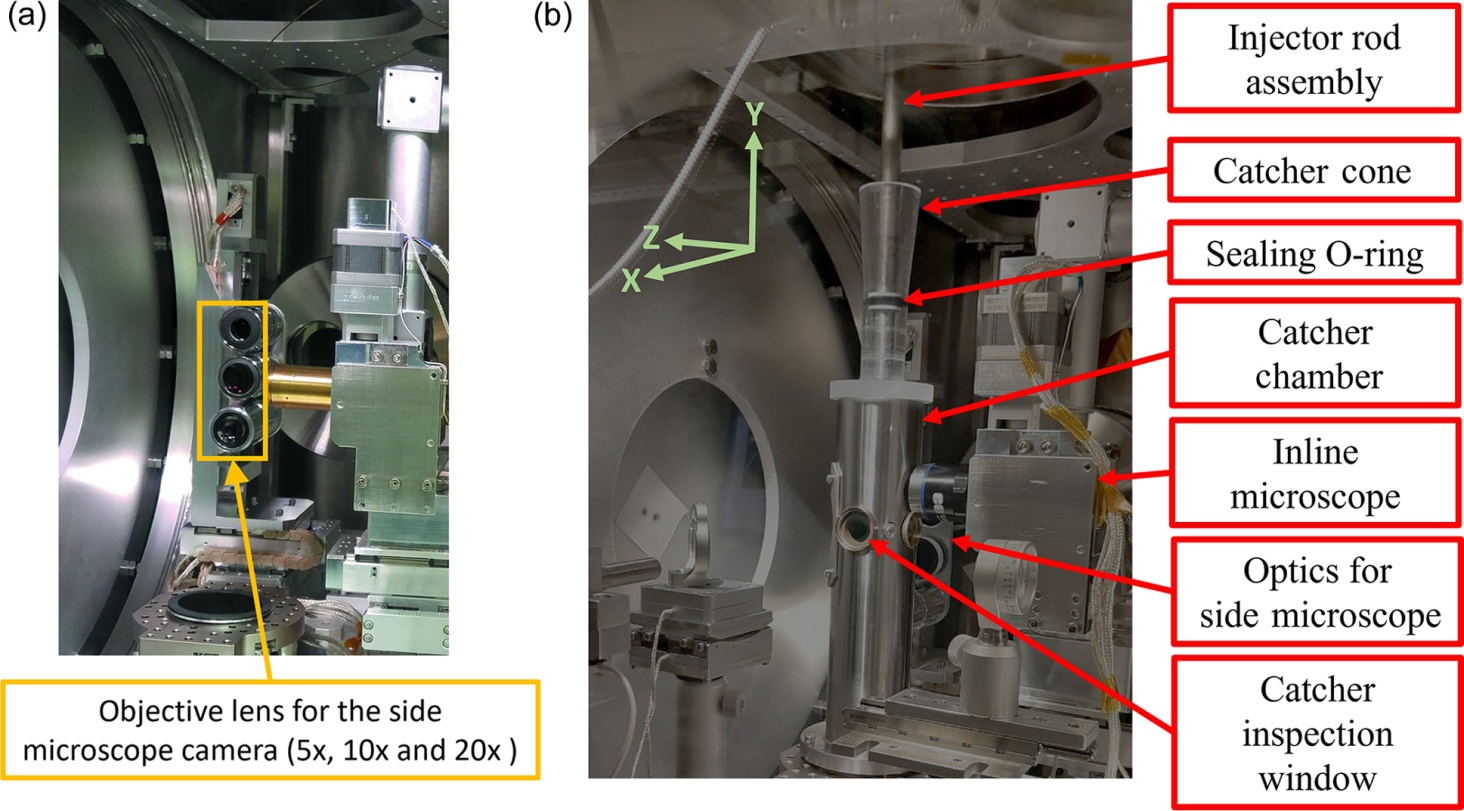
(*a*) Three objective lenses (5×, 10× and 20×) together with the camera attached to the side-view microscope, located along the *X* axis of the sample delivery setup. This microscope is used to monitor and align the liquid jet with the X-ray beam focus (the interaction point) during the experiment and is also the primary means used to capture images of the liquid jet during the experiment. (*b*) Portion of the vacuum chamber containing the nozzle and optical image capture setup shown in (*a*). The coordinate system for the SPB/SFX sample delivery setup is displayed in the top left-hand corner.

The current algorithm is capable of calculating jet re-alignment positions only along the *Z* axis, which is parallel to the beam propagation direction (see Fig. 2 ▸ for details). Assuming any straight lines within the image are associated with the liquid jet, the jet angle may be calculated directly from their slope, which is typically measured with respect to the initial jet position. An analysis of the jet stability can then be carried out on the basis of the change in jet angle as a function of time (Kim & Yoon, 2009 ▸). If the movement of the jet is greater than a predetermined set point then a change in motor position is required, and the *Z* axis motor can be adjusted to realign the position of the jet in the plane parallel to the optical path of the beam. Within the experiment, movements of the jet are classified into two broad categories: either momentary fluctuations in jet position or systematic drift. The former case does not necessarily require a motor move in order to correct the position since the jet will often move back into alignment again and remain there for a sufficient period of time such that the experiment can proceed. However, if the jet is constantly moving out of alignment with respect to the X-ray beam or there is a systematic drift in the position of the jet, then correction of the jet position or, in the worst case, cleaning or replacement of the nozzle may be required. Note that in the current experiment only the output of the side microscope was analysed with no automated motor moves implemented. The integration of this algorithm with all three motors supporting the injector nozzle (*i.e.X* and *Y* in addition to the current *Z* alignment) enabling continuous feedback will form the basis for the next stage in development.

### 2.2. Experimental setup within the sample delivery chamber at the EuXFEL SPB/SFX instrument

Fig. 2 ▸ shows the experimental setup used for liquid sample delivery at the SPB/SFX instrument at the EuXFEL. The side-view microscope [Fig. 2 ▸(*a*)] is aligned perpendicular to the axis of the X-ray beam and the liquid jet and is focused on the region of interaction where the beam is incident on the liquid jet (Patel *et al.*, 2022 ▸). The microscope is used to align the liquid jet nozzle [Fig. 2 ▸(*b*)] and the X-ray beam at the interaction point. The *X* axis refers to the horizontal direction and the *Y* axis the vertical direction in the plane perpendicular to the direction of propagation (*Z* axis).

Currently, the liquid jet alignment process is performed manually by an instrument scientist. The alignment process requires bringing the jet to the focal point of the X-ray beam in both the tangential (*X* axis) direction and the horizontal (*Z* axis) position (*i.e.* the X-ray focal plane). The side-view microscope enables continuous visual monitoring of jet stability during the experiment. For a detailed description of the SPB/SFX sample delivery setup the reader is referred to the literature (Patel *et al.*, 2022 ▸; Schulz *et al.*, 2019 ▸); in addition, Fig. S3 presents a schematic diagram of the sample delivery chamber. An inline microscope is also used for aligning the liquid jet to the X-ray beam. However, since the inline microscope can increase background scattering, it is typically removed from the beam path after the initial alignment. The inline camera system uses a 45° mirror with a 2 mm-diameter centre bore, intended to allow the X-ray beam to pass through (Schulz *et al.*, 2019 ▸). However, despite the large bore diameter relative to the beam size, the beam tails can clip the edges of the bore, generating a detectable background scattering signal at XFEL intensities, which interferes with the detection of the sample signal. Practically, the side microscope then becomes the primary diagnostic imaging source during the XFEL experiment.

Another motivation for the current project was the integration of our machine-vision-based jet alignment algorithm with the existing software and hardware components at the SPB/SFX instrument (Mills *et al.*, 2020 ▸; Mancuso *et al.*, 2013 ▸). The instrument equipment, including motors and pumps, is controlled via the Karabo system (Hauf *et al.*, 2019 ▸). This control system offers a Python-based application programming interface that enables users to interact with motors and perform image processing in real time.

A typical experimental setup used for liquid sample delivery at the SFX/SPB instrument consists of a nozzle–injector assembly, an *XYZ* stage for alignment and an sCMOS camera (the detector for the side-view microscope) usually oriented perpendicular to the direction of beam propagation (shown in Fig. 2 ▸) (Schulz *et al.*, 2019 ▸). The nozzle–injector assembly is suspended inside this chamber which is kept at a vacuum pressure of less than 10^−4^ mbar during experiments. The *XYZ* stage, which controls the position of the injector rod assembly, comprises three stepper motors, one for each axis. The rubber O-ring forms a seal for the catcher chamber and a pivoting point for the injector rod. The light source for illumination inside the sample chamber used for image acquisition is a nanosecond laser with a typical duration of 5–7 ns (Koliyadu *et al.*, 2022 ▸). The illuminating laser is triggered for stroboscopic illumination after a given X-ray pulse within the train, usually the second or third pulse, to capture the gap created by the X-ray-induced explosion and the jet recovery from pulse to pulse (to validate jet replenishment between pulses) (Koliyadu *et al.*, 2022 ▸). The field of view of the camera encompasses the tip of the nozzle and the entire length of the liquid jet when using a 10× objective. The incident X-ray photon energy was set to 9.3 keV, with an average pulse energy of 1.5 mJ pulse^−1^ (Mancuso *et al.*, 2019 ▸).

In a typical pump–probe experiment, the pump–probe laser is not triggered simultaneously with the illumination laser, avoiding compatibility issues. However, if they are triggered together, a change in the dynamic range of the image between frames can affect the thresholding of the jet alignment algorithm. To address this, Otsu’s method is used to dynamically adjust the threshold value, maintaining algorithm performance. The pump–probe laser has wavelengths of 800 or 1030 nm, with repetition rates from 10 to 4.5 MHz. By contrast, the wavelength of the illumination laser is within the visible range and is adjustable to prevent unwanted excitation within the sample.

### 2.3. Sample and jetting conditions

The three nozzles used here for generating the liquid jets were the GDVN with liquid aperture sizes of 75 µm (gas orifices of 60 µm) and 100 µm (gas orifices of 75 µm), and the DFFN with a liquid aperture size of 75 µm (gas orifices of 70 µm) (Vakili *et al.*, 2020 ▸, 2022 ▸). All three nozzles are commonly used for liquid jet experiments at the SPB/SFX instrument of the EuXFEL (Vakili *et al.*, 2022 ▸). Further details of the nozzle designs are available via GitHub (Vakili & Otte, 2021 ▸). The jet speeds selected for sample jetting fall into two categories corresponding to the two most used repetition rates, 1.1 and 0.5 MHz. Sample replenishment at the 1.1 MHz repetition rate requires a minimum jet speed of 45 m s^−1^ (Vakili *et al.*, 2022 ▸). To test a variety of instability conditions related to this case, jet speeds of 40 and 50 m s^−1^ were also used. For the 0.5 MHz repetition rate, a jet speed of 25 m s^−1^ is the minimum (Vakili *et al.*, 2022 ▸). Though ideally we would have used jet speeds of 20 m s^−1^ as well as 30 m s^−1^ to also measure above and below the speed of 25 m s^−1^, the target jet speed of 20 m s^−1^ was found to be incompatible with the nozzles used in this experiment as a jet could not be established and therefore these data could not be included. We note that the purpose is not to test various jetting conditions but rather to test the performance of the algorithm in assessing the physical characteristics of liquid jets for the most used cases at the SPB/SFX instrument.

The samples used here for testing and analysis were water, storage buffer and buffer containing lysozyme crystals in storage buffer. The lysozyme crystals were grown with diameters of <1, 2–4 and 4–6 µm – confirmed via optical microscopy. Lysozyme crystals were selected as a model system due to the ease with which they can be produced, and their diameters controlled. These crystal sizes are also routinely used for SFX experiments. Lysozyme crystals were prepared by rapid vertexing of a 1:1 ratio aqueous solution of lysozyme [100 mg ml^−1^ in 50 m*M* NaOAc (pH 3.5)], filtered with a 0.2 µm syringe filter to remove impurities and crystallization solution [0.1 *M* NaOAc (pH 3.5), 5% PEG 6000 (*v*/*v*), 3.2 *M* NaCl] at 20°C. After crystallization, the remaining solution was exchanged to a storage buffer [50 m*M* NaOAc (pH 3.5), 1.7 *M* (10%) NaCl] by centrifugation at 200*g*. The crystal slurry was passed through a Nylon mesh gravity filter (20 µm wide meshes, CellTrics) to remove the largest particles before use which helps to prevent the nozzles clogging. Table S1 of the supporting information gives the jetting conditions and their corresponding nozzles.

## 3. Results and discussion

The investigation of a range of samples and jetting parameters allows us to test the fidelity of the algorithm’s ‘stability factor’ calculation using liquid jets with various combinations of jetting parameters. This factor can be used to help guide the decision-making process for managing jetting and determining when manual intervention is required in the experiment. To enable the full range of samples to be measured during the available beam time, each sample was run for a total of 120 s with data collected at 10 Hz. Note that the 10 Hz image acquisition rate, while sufficient to characterize jet stability, imposes a sampling limitation. This results in discrete and potentially misrepresentative snapshots of the jet motion compared with higher-frequency quasi-continuous tracking. Consequently, it becomes challenging to accurately follow the jet’s drift and precession if they occur at a rate faster than this 10 Hz sample rate. Nevertheless, this suffices for in-practice measurements at the EuXFEL with a 10 Hz pulse train rate

After initialization, the liquid jet was allowed to stabilize for 60 s. The optical microscopy data were then analysed for a further 60 s using our machine-vision algorithm. This amount of data and the jetting duration were sufficient to characterize both ‘short-’ and ‘long-term’ jet instabilities. Here, short-term instabilities in the liquid jet are characterized by constant lateral movements occurring at very high rates (up to the pulse train frequency of the EuXFEL, *e.g.* 10 Hz) and often result in ‘sputtering’. These typically cannot be corrected through nozzle position adjustments. In contrast, longer-term instabilities, which occur over several seconds to tens of seconds, can be corrected using nozzle adjustment feedback and benefit from jet realignment.

### 3.1. Real-time analysis of hit rates for liquid jets

**Figure 3:**
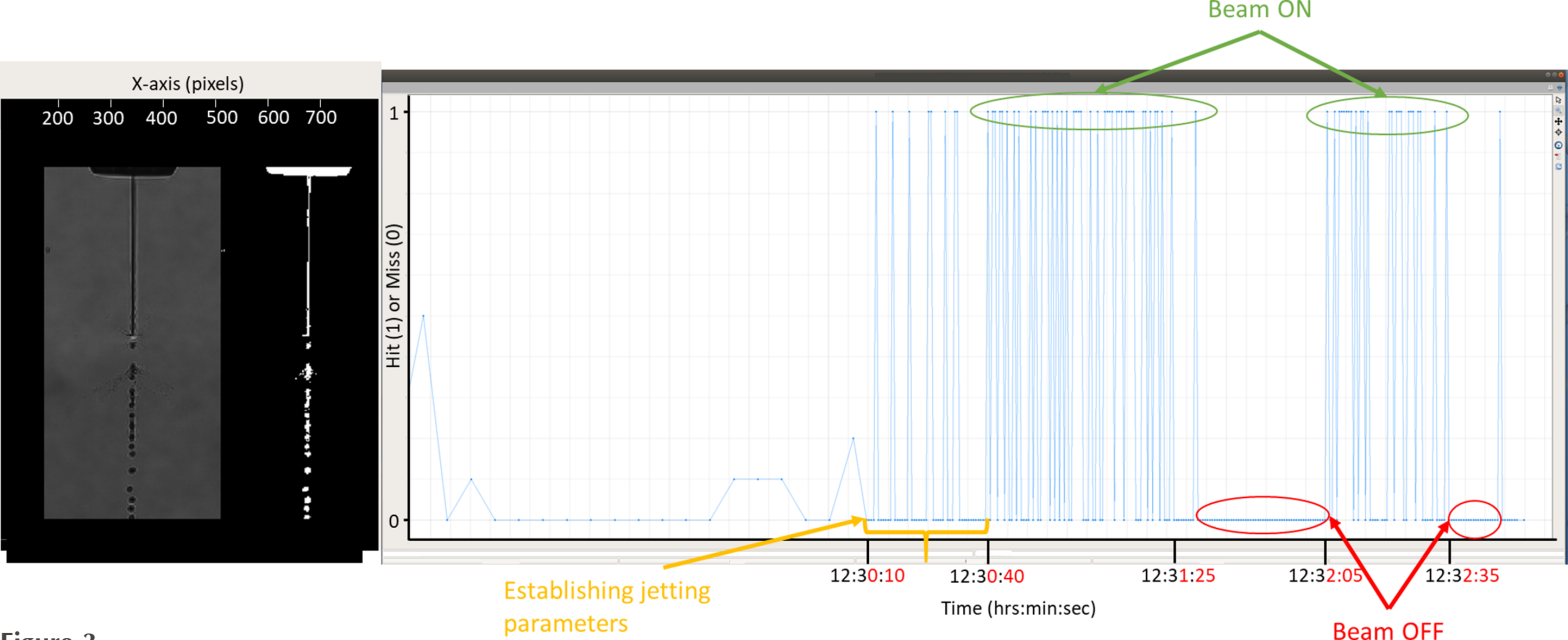
Screenshot of the Karabo machine-vision algorithm GUI illustrating real-time automated overlap detection running at the SPB/SFX instrument. The image on the left shows the optical microscopy image and the corresponding binarized image of the liquid jet after ROI cropping. The graph on the right shows the algorithm output which classifies the image of the liquid jet acquired from the side microscope as either a ‘hit’ or a ‘miss’ (represented as a ‘1’ or ‘0’, respectively). Prior to establishing the jet at 12:30:10, the Karabo control system sends a ‘dummy’ data stream as input for the GUI, hence the appearance of fractional values between 0 and 1.

The machine-vision algorithm was previously applied offline to optical microscopy data to validate its performance in terms of classifying ‘hits’ and ‘misses’ against well characterized datasets which consisted of ultra-pure water being jetted (Patel *et al.*, 2022 ▸). The extension of the algorithm to real-time analysis of liquid jet–XFEL beam overlap as part of the present work required the integration of the algorithm into Karabo. A screenshot of the Karabo GUI for the alignment algorithm which displays the cropped version of the original image (acquired via the side-view microscopy camera setup) and the corresponding binarized image is shown Fig. 3 ▸. On the right is a graph which displays the output of the hit classification of the liquid jet images: ‘1’ means ‘hit’, *i.e.* the liquid jet and beam intersect; and ‘0’ means a ‘miss’, *i.e.* the XFEL beam misses the jet.

The classification algorithm (identifying a ‘hit’ or ’miss’) was tested during regular user beam time at the EuXFEL, this experiment was carried out independently of the jet angle statistical analysis performed in this paper. In this experiment, a DFFN75 nozzle was used to inject crystals during user beam time into the X-ray beam. The graph on the right-hand side in Fig. 3 ▸ represents the data captured during the first 175 s (2 min, 55 s) of beam time. The section of the graph starting from the yellow arrow labelled ‘establishing jetting parameters’ lasting for 30 s was measured during initial stabilization of the jet, while parameters including liquid flow rate and gas pressure were still being optimized. Consequently, the hit rate is lower during this time period. In the sections when the beam is on it is evident that the jet position is still relatively unstable as the system records a miss for significant portions of time. As expected, once the beam is switched off, the hit rate drops to zero and no ‘false positives’ were recorded. During the experiment, the algorithm analysed data in real time without any issues or noticeable lag in the Karabo control system or GUI. This marks a crucial first step towards automating the process of aligning the liquid jet with the beam. The next step in implementing the algorithm was to conduct an in-depth stability analysis of the liquid jet, going beyond simply monitoring the XFEL beam-jet overlap. The initial step in this analysis involved calculating and analysing the jet angle relative to the nozzle, which serves as a key metric for quantifying jet stability.

### 3.2. Analysis of liquid jet angular displacement

**Figure 4:**
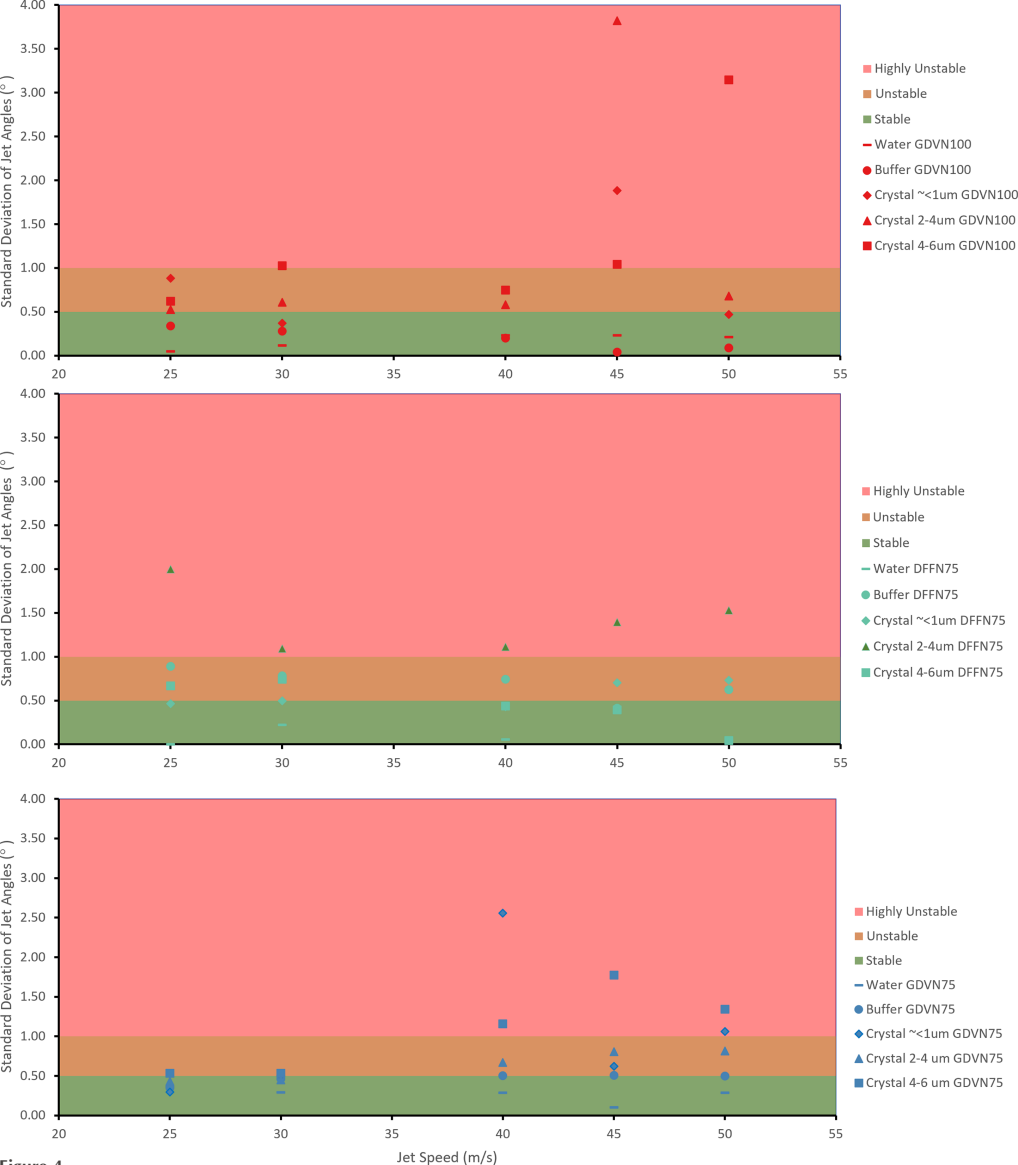
Scatter plots of the standard deviation (°) of the jet angles for each of the three categories in the dataset, with each data point involving the analysis of 600 side microscopy images. A higher value of standard deviation reflects higher instability, with a standard deviation of 1° corresponding to the transition from ‘unstable’ to ‘highly unstable’ jet.

Fig. 4 ▸ plots the standard deviation (°) of the liquid jet angle, where the standard deviation represents the spread in the jet angle evaluated over 600 frames of data, as determined by our algorithm for all samples tested across a range of jetting speeds. Each single data point in Fig. 4 ▸ represents the analysis of the jet angle by our algorithm over 600 images (60 s) of data collection.

We classified the jet behaviour as ‘stable’, ‘unstable’ and ‘highly unstable’ based on the experience of jetting experts and instrument scientists accumulated during more than 5 years of operation of liquid jets for megahertz repetition rate experiments. For each of these classifications the standard deviation value of liquid jet angle was measured. For the ‘stable’ category the observed standard deviation was less than 0.5°. For the ‘unstable’ category the observed standard deviation was between 0.5 and 1°. For the ‘highly unstable’ category the observed standard deviation was greater than 1°. These values correspond to the green, amber and red shaded areas of the graphs shown in Fig. 4 ▸, respectively. Note that these values were determined specifically for the dataset collected in this experiment; they represent limits which can be adjusted according to the dataset and the operator’s observations in order to quantify the stability of a liquid jet.

In the ‘stable’ case (jet angle standard deviation < 0.5°), the displacement of the jet is typically between 0 and 1°, and although the sample stream may move outside of the XFEL interaction region, it would be for no more than a few consecutive frames at a time. This results in a shift of interaction points which will be out of focus with respect to the detector before returning. Note that when the liquid jet moves outside the interaction region along the *Z* axis, the jet would still interact with the beam; however, the sample-to-detector distance will be changed, potentially affecting data processing for these frames. Stable jets would not typically require any remedial action by the instrument scientist to bring the jet back into alignment and some motion is to be expected as unavoidable.

In the ‘unstable’ (0.5° < standard deviation < 1.0°) case, the maximum angular displacement of the jet from its aligned position was observed to be ±2°. This behaviour occurring continuously at random intervals can result in the jet missing the XFEL beam for a substantial portion of time. If such behaviour is a longer-term deviation, movement (realignment) of the injector nozzle can be used to maintain the jet and X-ray alignment. For short-term instabilities, adjustment of gas pressure or liquid flow rate would be recommended and may be sufficient to maintain an effective X-ray–jet overlap during data collection.

Finally, in the ‘highly unstable’ (standard deviation > 1.0°) case, jets exhibit either drastic random motion or early break-up of the jet resulting in a jet length much shorter than is typically observed for stable jets. Jets within this category would require immediate intervention by the instrument scientists, which would often involve either cleaning or replacing the nozzle. For example, to demonstrate such a level of instability, conditions were selected to guarantee a highly unstable jet. Use of GDVN100 at higher jet speeds of 45 m s^−1^ produces the desired jet behaviour, as these nozzles are designed for lower jet speeds. This enabled us to test the ability of the algorithm to classify highly unstable jets.

Though jet length is another potential metric for stability, its usefulness is limited by the presence of a gap at the interaction region. When properly aligned, the jet length is always equal to the distance between the nozzle tip and the interaction point, making it a less informative parameter. Therefore, the standard deviation of the jet angle along the *Z* axis was found to be a more viable metric for assessing jet stability across the various conditions tested. However, for offline testing without the presence of the jet explosion, jet length can be a valuable quantitative metric for characterizing nozzles. It can also help to inform the optimal jetting parameters for a given sample prior to the data collection stage of the experiment.

The results in Fig. 4 ▸ may provide insights into when the current algorithm could be employed to correct the jet position or decide when to clean or replace the nozzle altogether. Additionally, the corresponding Videos S1 and S2 of the supporting information confirm that a highly unstable jet is characterized by rapid back-and-forth oscillations of the liquid stream. Such cases generally cannot be remedied by the development of an automatic realignment algorithm.

### 3.3. Jet drift versus rapid oscillation

**Figure 5:**
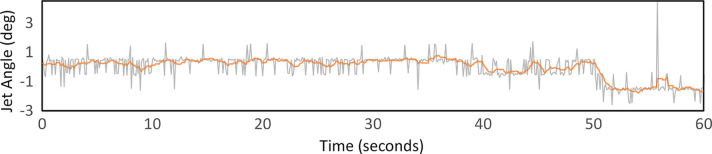
Graph showing representative jet angles for a buffer and sample containing 1 µm crystals formed using a DFFN with a 75 µm aperture at a jet speed of 45 m s^−1^. The plot shows the raw data in grey, and data averaged over 1 s in orange. The drift region indicates the drift in the jet angle which results in misalignment of the jet with respect to the beam. ‘Peaks’ indicate momentary instabilities in the liquid jet that cause the jet to swing laterally for a moment, evident by a drastic change in the jet angle.

During liquid jetting, the jet will typically ‘jitter’ back and forth. Provided these motions are small on the scale of the X-ray focus, there is no action required on the part of the instrument scientist. However, often the jet will undergo a period of constant motion in one direction before settling into a new stable position. If the new position places the jet outside the interaction region of the XFEL beam, then the instrument scientist will need to manually realign the jet by moving the *XYZ* positioner for the injector rod. The other feature worth noting is that the jet briefly ‘flicks’ from one position to another before returning to its original position. This type of behaviour will manifest as a discrete peak in the plot of jet angle versus time. Fig. 5 ▸ presents a representative plot illustrating all three types of behaviour. A moving average window of 10 frames (1 s) was used to highlight the trends in the data – shown by the orange line overlaying the raw data. The short-term jitter in the angle can be seen occurring between 0 and 50 s. A period of jet angle drift is highlighted at around the 50 s mark and a short-term large angle variation can be seen at about 56 s. Fig. S2 provides a comparison with moving average window sizes of 5 and 20 frames.

### 3.4. Jet statistical analysis

**Figure 6:**
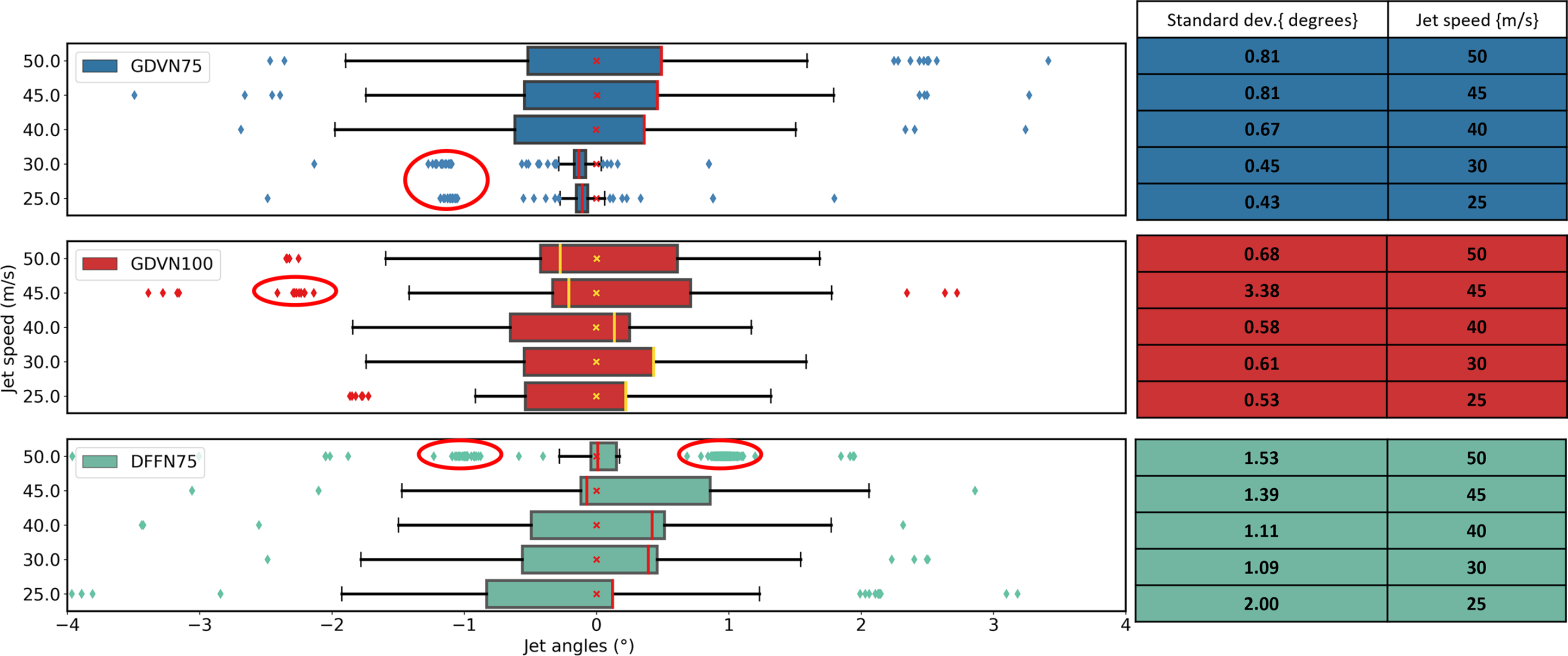
Box plots summarizing jet angle data for the three different nozzle types (GDVN – 75 µm aperture, GDVN – 100 µm aperture and DFFN – 75 µm aperture), indicated in the top left-hand corner of the plots. The samples used to generate these data were the 2–4 µm lysozyme crystal sample. The table on the right-hand side shows the standard deviation (°) in the jet angle calculated over 600 images (*i.e.* 60 s of data collection). In the box-and-whisker plots, the lengths of the box and the whiskers represent the jet angle variation between the lower and upper quartiles (inter quartile range – IQR) and the minimum and maximum data values (excluding outliers), respectively. The position of the median value, indicated by the vertical line bis­ecting the box, indicates the skewness of the data (*i.e.* the further from the centre of the box, the more skewed the histogram). The outliers marked by the isolated data points were defined on the basis of the fact that they fall outside of the statistical limit of 1.5 × IQR.

Some further insights into the behaviour of the liquid jet over time can be gained from the ‘box-and-whisker’ plots given in Fig. 6 ▸. These show that the distribution of angles is not well represented by a Gaussian distribution, as the data are skewed and have a significant number of outliers. Accordingly, we opted to use the standard deviation as the preferred metric for stability rather than the inter-quartile range (IQR), which here is similar across all the nozzle types used.

From the individual data points in all datasets shown in Fig. 6 ▸, it can be observed that the jet angle data frequently form clusters or sub-groups (circled regions in Fig. 6 ▸), which may indicate one of two conditions: firstly, a jet which has continuous axial precession around the vertical *Y* axis may be considered dynamically unstable and cannot be corrected by realignment (see Video S1). Secondly, these clusters may indicate reoccurring angular displacement at a predictable recurrent angle, which could be corrected using an automated monitoring and alignment system. We have demonstrated that we can discriminate between these two cases using the standard deviation of the jet angle. The standard deviation values illustrated in Fig. 4 ▸ and the tables on the right side of Fig. 6 ▸ are consistent with the trend, which is that the smaller the standard deviation of the jet angle, the higher the observed jet stability.

In order to validate the effectiveness of our algorithm with real-world liquid sample delivery methods, three commonly used nozzles were applied: GDVN75, GDVN100 and DFFN75. In this study, we found that our algorithm can provide quantification of stability for each jetting condition. These quantification values of stability provide thresholds for decision making in preparation for and during data collection.

Another advantage of determining the jet angle during the experiment is that it can identify which jetting conditions are compatible with a particular combination of sample and nozzle types. This tool enables the real-time observation of jet stability as a number of jetting condition parameters are varied, such as liquid and gas pressures as well as a range of different nozzles. With this capability and other metrics, additional experiments would allow one to explore the conditions driving the origin of any particular instability. The stability metric presented here quantifies the observed behaviour under the conditions investigated, enabling a number of potential new applications. First, the sample is jetted under a variety of different conditions and can be assigned a performance score which will help determine best possible jetting parameters for that sample. Second, during the experiment the algorithm can be programmed to monitor jet behaviour in real time, correct for jet drifts and warn instrument scientists if the jet instability increases above a set limit. Both applications can help to improve sample delivery efficiency and provide a diagnostic method for trouble shooting and most importantly automating the tedious task of continuous manual monitoring of the liquid jets during the experiment.

Another potential use of this algorithm is for offline testing of various combinations of jetting parameters before the experiment, which may help identify the most stable jet parameters for data collection for a given sample. This could reduce the time needed to establish stable jetting during the experiment, thus minimizing sample wastage and the beam time required. For example, it may be possible to use this algorithm as a diagnostic tool for assessing a batch of 3D-printed nozzles prior to beam time. This tool could provide a quantifiable value for identifying potentially defective nozzles in a production batch. Additionally, it might offer insights into alternative nozzle designs for future developments, allowing for an extensive testing regime to quantify nozzle performance prior to an experiment.

It should also be emphasized that, while these observations hold for the specific set of experiments carried out here, they are not necessarily reflective of the general behaviour and performance of these nozzles across all sample and jetting conditions. Nevertheless, our methods do allow for systematic investigation for any given sample(s), buffer(s), nozzle(s) and jetting condition(s). Each specific combination of nozzle and sample type corresponds to a single experimental run. The samples were jetted in the order of <1 µm crystals, followed by 2–4 µm crystals and then finally 4–6 µm crystals.

## 4. Conclusions

On the basis of the comprehensive review of the data presented herein, it can be concluded that our algorithm provides a means of monitoring variations in the liquid jet angle, which in turn is an indication of jet stability. This metric can be used to guide automatic realignment and inform operators when either cleaning or replacement might be required. The correspondence between standard deviation and jet stability was further confirmed by comparing expert examination of videos of the jets, which revealed a positive correlation, regardless of the sample or nozzle type used.

Given the versatility of a machine-vision-based system, this algorithm can be used for various tasks. A primary example of such use would be to investigate the influence of crystal size and jet speed, alone and/or in combination, to form a stable liquid jet suitable for megahertz SFX. To further investigate these trends, additional data need to be incorporated that cover a broader range of jetting parameters and nozzle types for liquid jet formation. Considering that the nozzle type and aperture size contribute to jet stability, the standard deviation of the jet angle may also provide insights into the effect of any irregularities during nozzle fabrication. This is another possible use for this algorithm. However, this also warrants further investigation, and no definitive conclusions can be drawn in this regard solely based on the data collected in this experiment. The algorithm can be used to test jetting parameters in offline testing to ascertain a viable combination of jetting parameters for a particular sample, which will help reduce issues with jetting during the experiment and could be used as a diagnostic tool for trouble shooting.

The algorithm can also be applied in other contexts, but it will necessitate modifications tailored to the specific requirements of each application. By utilizing the statistical data analysis generated by our real-time jet analysis algorithm, a vision-based jet alignment system could be developed to automate the alignment process and provide an early warning system for detecting unstable liquid jets. One such potential use of this algorithm would be to help monitor for issues such as detecting icicles during jetting, which can permanently damage the detector pixel modules. Another application would be to use the output from the machine-vision algorithm to generate 2D plots of image metrics directly in the Karabo control system. This information could then be used by instrument scientists to detect a range of critical issues including sample leakage, resulting in sudden liquid/gas flow decrease, monitoring of the current volume of the sample in the reservoir, sudden or slow change in gas pressure, and many other aspects of jetting.

Our algorithm for determining jet angle during experiments offers multiple uses. First, it identifies compatible jetting conditions for specific samples and nozzles by enabling real-time observation of jet stability under varied parameters. This allows optimization of jetting conditions, real-time monitoring, correction of drifts and warnings for instability, thus improving sample delivery efficiency and automating manual monitoring. Additionally, it can be used offline to test jetting parameters before experiments, reducing setup time and sample wastage. Finally, it also serves as a diagnostic tool for assessing 3D-printed nozzles, identifying defects and informing alternative designs through pre-testing.

The use of this tool is also not limited to the EuXFEL. Any other light source facility with a comparable setup can implement this system with some minor modifications. This system can also be adapted for monitoring other jet-based sample delivery methods. However, depending on the sample delivery method, the logic and feature-finding would need to be adjusted accordingly. For example, ensuring stability when tracing a droplet injector would require tuning the Hough lines transform to track a series of sections in a straight line or replacing the Hough line transform with line fitting algorithms.

The algorithm discussed in this study has been successfully implemented at the SPB/SFX instrument of the EuXFEL and is now readily accessible for users seeking to monitor the stability and characteristics of their liquid jets. An additional approach to gauge jet stability, such as evaluating liquid jet length alongside jet angle, could enhance the level of detail available from any jet behaviour analysis. This work paves the way for future advancements in machine-vision-based analysis of optical microscopy images of liquid jets, offering many promising avenues for further research and development. This includes the ability to perform quantitative analysis for jetting studies to help refine jetting conditions for a specific sample, which would help reduce sample consumption during beam time. Use of the tool during an experiment can also assist the operators in monitoring jetting more efficiently, as it can be used as a diagnostic tool to troubleshoot jetting issues on the go. Another possible future application – following additional research and the collection of a comprehensive dataset to characterize – would be to investigate the effects of sample size and jet speed on the stability of a jet.

## Supplementary Material

Supporting figures, tables and captions to videos. DOI: 10.1107/S1600576724009853/te5136sup1.pdf

Video S1. DOI: 10.1107/S1600576724009853/te5136sup2.mp4

Video S2. DOI: 10.1107/S1600576724009853/te5136sup3.mp4

Video S3. DOI: 10.1107/S1600576724009853/te5136sup4.mp4

## References

[bb1] Barends, T. R. M., Stauch, B., Cherezov, V. & Schlichting, I. (2022). *Nat. Rev. Methods Primers*, **2**, 59. 10.1038/s43586-022-00141-7 PMC9833121 36643971

[bb30] Berntsen, P., Hadian Jazi, M., Kusel, M., Martin, A. V., Ericsson, T., Call, M. J., Trenker, R., Roque, F. G., Darmanin, C. & Abbey, B. (2019). *Rev. Sci. Instrum.* **90**, 085110. 10.1063/1.5104298 31472610

[bb2] Beyerer, J., Puente León, F. & Frese, C. (2016). *Machine Vision. Automated Visual Inspection: Theory, Practice and Applications*, 1st ed. Berlin, Heidelberg: Springer.

[bb3] Beyerlein, K. R., Adriano, L., Heymann, M., Kirian, R., Knoška, J., Wilde, F., Chapman, H. N. & Bajt, S. (2015). *Rev. Sci. Instrum.* **86**, 125104. 10.1063/1.4936843 26724070

[bb4] Chapman, H. N., Fromme, P., Barty, A., White, T. A., Kirian, R. A., Aquila, A., Hunter, M. S., Schulz, J., DePonte, D. P., Weierstall, U., Doak, R. B., Maia, F. R. N. C., Martin, A. V., Schlichting, I., Lomb, L., Coppola, N., Shoeman, R. L., Epp, S. W., Hartmann, R., Rolles, D., Rudenko, A., Foucar, L., Kimmel, N., Weidenspointner, G., Holl, P., Liang, M., Barthelmess, M., Caleman, C., Boutet, S., Bogan, M. J., Krzywinski, J., Bostedt, C., Bajt, S., Gumprecht, L., Rudek, B., Erk, B., Schmidt, C., Hömke, A., Reich, C., Pietschner, D., Strüder, L., Hauser, G., Gorke, H., Ullrich, J., Herrmann, S., Schaller, G., Schopper, F., Soltau, H., Kühnel, K.-U., Messerschmidt, M., Bozek, J. D., Hau-Riege, S. P., Frank, M., Hampton, C. Y., Sierra, R. G., Starodub, D., Williams, G. J., Hajdu, J., Timneanu, N., Seibert, M. M., Andreasson, J., Rocker, A., Jönsson, O., Svenda, M., Stern, S., Nass, K., Andritschke, R., Schröter, C.-D., Krasniqi, F., Bott, M., Schmidt, K. E., Wang, X., Grotjohann, I., Holton, J. M., Barends, T. R. M., Neutze, R., Marchesini, S., Fromme, R., Schorb, S., Rupp, D., Adolph, M., Gorkhover, T., Andersson, I., Hirsemann, H., Potdevin, G., Graafsma, H., Nilsson, B. & Spence, J. C. H. (2011). *Nature*, **470**, 73–77.

[bb5] Cheng, R. K. Y. (2020). *Crystals*, **10**, 215.

[bb6] Decking, W., Abeghyan, S., Abramian, P., Abramsky, A., Aguirre, A., Albrecht, C., Alou, P., Altarelli, M., Altmann, P., Amyan, K., Anashin, V., Apostolov, E., Appel, K., Auguste, D., Ayvazyan, V., Baark, S., Babies, F., Baboi, N., Bak, P., Balandin, V., Baldinger, R., Baranasic, B., Barbanotti, S., Belikov, O., Belokurov, V., Belova, L., Belyakov, V., Berry, S., Bertucci, M., Beutner, B., Block, A., Blöcher, M., Böckmann, T., Bohm, C., Böhnert, M., Bondar, V., Bondarchuk, E., Bonezzi, M., Borowiec, P., Bösch, C., Bösenberg, U., Bosotti, A., Böspflug, R., Bousonville, M., Boyd, E., Bozhko, Y., Brand, A., Branlard, J., Briechle, S., Brinker, F., Brinker, S., Brinkmann, R., Brockhauser, S., Brovko, O., Brück, H., Brüdgam, A., Butkowski, L., Büttner, T., Calero, J., Castro-Carballo, E., Cattalanotto, G., Charrier, J., Chen, J., Cherepenko, A., Cheskidov, V., Chiodini, M., Chong, A., Choroba, S., Chorowski, M., Churanov, D., Cichalewski, W., Clausen, M., Clement, W., Cloué, C., Cobos, J. A., Coppola, N., Cunis, S., Czuba, K., Czwalinna, M., D’Almagne, B., Dammann, J., Danared, H., de Zubiaurre Wagner, A., Delfs, A., Delfs, T., Dietrich, F., Dietrich, T., Dohlus, M., Dommach, M., Donat, A., Dong, X., Doynikov, N., Dressel, M., Duda, M., Duda, P., Eckoldt, H., Ehsan, W., Eidam, J., Eints, F., Engling, C., Englisch, U., Ermakov, A., Escherich, K., Eschke, J., Saldin, E., Faesing, M., Fallou, A., Felber, M., Fenner, M., Fernandes, B., Fernández, J. M., Feuker, S., Filippakopoulos, K., Floettmann, K., Fogel, V., Fontaine, M., Francés, A., Martin, I. F., Freund, W., Freyermuth, T., Friedland, M., Fröhlich, L., Fusetti, M., Fydrych, J., Gallas, A., García, O., Garcia-Tabares, L., Geloni, G., Gerasimova, N., Gerth, C., Geßler, P., Gharibyan, V., Gloor, M., Głowinkowski, J., Goessel, A., Gołębiewski, Z., Golubeva, N., Grabowski, W., Graeff, W., Grebentsov, A., Grecki, M., Grevsmuehl, T., Gross, M., Grosse-Wortmann, U., Grünert, J., Grunewald, S., Grzegory, P., Feng, G., Guler, H., Gusev, G., Gutierrez, J. L., Hagge, L., Hamberg, M., Hanneken, R., Harms, E., Hartl, I., Hauberg, A., Hauf, S., Hauschildt, J., Hauser, J., Havlicek, J., Hedqvist, A., Heidbrook, N., Hellberg, F., Henning, D., Hensler, O., Hermann, T., Hidvégi, A., Hierholzer, M., Hintz, H., Hoffmann, F., Hoffmann, M., Hoffmann, M., Holler, Y., Hüning, M., Ignatenko, A., Ilchen, M., Iluk, A., Iversen, J., Iversen, J., Izquierdo, M., Jachmann, L., Jardon, N., Jastrow, U., Jensch, K., Jensen, J., Jeżabek, M., Jidda, M., Jin, H., Johansson, N., Jonas, R., Kaabi, W., Kaefer, D., Kammering, R., Kapitza, H., Karabekyan, S., Karstensen, S., Kasprzak, K., Katalev, V., Keese, D., Keil, B., Kholopov, M., Killenberger, M., Kitaev, B., Klimchenko, Y., Klos, R., Knebel, L., Koch, A., Koepke, M., Köhler, S., Köhler, W., Kohlstrunk, N., Konopkova, Z., Konstantinov, A., Kook, W., Koprek, W., Körfer, M., Korth, O., Kosarev, A., Kosiński, K., Kostin, D., Kot, Y., Kotarba, A., Kozak, T., Kozak, V., Kramert, R., Krasilnikov, M., Krasnov, A., Krause, B., Kravchuk, L., Krebs, O., Kretschmer, R., Kreutzkamp, J., Kröplin, O., Krzysik, K., Kube, G., Kuehn, H., Kujala, N., Kulikov, V., Kuzminych, V., La Civita, D., Lacroix, M., Lamb, T., Lancetov, A., Larsson, M., Le Pinvidic, D., Lederer, S., Lensch, T., Lenz, D., Leuschner, A., Levenhagen, F., Li, Y., Liebing, J., Lilje, L., Limberg, T., Lipka, D., List, B., Liu, J., Liu, S., Lorbeer, B., Lorkiewicz, J., Lu, H. H., Ludwig, F., Machau, K., Maciocha, W., Madec, C., Magueur, C., Maiano, C., Maksimova, I., Malcher, K., Maltezopoulos, T., Mamoshkina, E., Manschwetus, B., Marcellini, F., Marinkovic, G., Martinez, T., Martirosyan, H., Maschmann, W., Maslov, M., Matheisen, A., Mavric, U., Meißner, J., Meissner, K., Messerschmidt, M., Meyners, N., Michalski, G., Michelato, P., Mildner, N., Moe, M., Moglia, F., Mohr, C., Mohr, S., Möller, W., Mommerz, M., Monaco, L., Montiel, C., Moretti, M., Morozov, I., Morozov, P., Mross, D., Mueller, J., Müller, C., Müller, J., Müller, K., Munilla, J., Münnich, A., Muratov, V., Napoly, O., Näser, B., Nefedov, N., Neumann, R., Neumann, R., Ngada, N., Noelle, D., Obier, F., Okunev, I., Oliver, J. A., Omet, M., Oppelt, A., Ottmar, A., Oublaid, M., Pagani, C., Paparella, R., Paramonov, V., Peitzmann, C., Penning, J., Perus, A., Peters, F., Petersen, B., Petrov, A., Petrov, I., Pfeiffer, S., Pflüger, J., Philipp, S., Pienaud, Y., Pierini, P., Pivovarov, S., Planas, M., Pławski, E., Pohl, M., Polinski, J., Popov, V., Prat, S., Prenting, J., Priebe, G., Pryschelski, H., Przygoda, K., Pyata, E., Racky, B., Rathjen, A., Ratuschni, W., Regnaud-Campderros, S., Rehlich, K., Reschke, D., Robson, C., Roever, J., Roggli, M., Rothenburg, J., Rusiński, E., Rybaniec, R., Sahling, H., Salmani, M., Samoylova, L., Sanzone, D., Saretzki, F., Sawlanski, O., Schaffran, J., Schlarb, H., Schlösser, M., Schlott, V., Schmidt, C., Schmidt-Foehre, F., Schmitz, M., Schmökel, M., Schnautz, T., Schneidmiller, E., Scholz, M., Schöneburg, B., Schultze, J., Schulz, C., Schwarz, A., Sekutowicz, J., Sellmann, D., Semenov, E., Serkez, S., Sertore, D., Shehzad, N., Shemarykin, P., Shi, L., Sienkiewicz, M., Sikora, D., Sikorski, M., Silenzi, A., Simon, C., Singer, W., Singer, X., Sinn, H., Sinram, K., Skvorodnev, N., Smirnow, P., Sommer, T., Sorokin, A., Stadler, M., Steckel, M., Steffen, B., Steinhau-Kühl, N., Stephan, F., Stodulski, M., Stolper, M., Sulimov, A., Susen, R., Świerblewski, J., Sydlo, C., Syresin, E., Sytchev, V., Szuba, J., Tesch, N., Thie, J., Thiebault, A., Tiedtke, K., Tischhauser, D., Tolkiehn, J., Tomin, S., Tonisch, F., Toral, F., Torbin, I., Trapp, A., Treyer, D., Trowitzsch, G., Trublet, T., Tschentscher, T., Ullrich, F., Vannoni, M., Varela, P., Varghese, G., Vashchenko, G., Vasic, M., Vazquez-Velez, C., Verguet, A., Vilcins-Czvitkovits, S., Villanueva, R., Visentin, B., Viti, M., Vogel, E., Volobuev, E., Wagner, R., Walker, N., Wamsat, T., Weddig, H., Weichert, G., Weise, H., Wenndorf, R., Werner, M., Wichmann, R., Wiebers, C., Wiencek, M., Wilksen, T., Will, I., Winkelmann, L., Winkowski, M., Wittenburg, K., Witzig, A., Wlk, P., Wohlenberg, T., Wojciechowski, M., Wolff-Fabris, F., Wrochna, G., Wrona, K., Yakopov, M., Yang, B., Yang, F., Yurkov, M., Zagorodnov, I., Zalden, P., Zavadtsev, A., Zavadtsev, D., Zhirnov, A., Zhukov, A., Ziemann, V., Zolotov, A., Zolotukhina, N., Zummack, F. & Zybin, D. (2020). *Nat. Photon.* **14**, 391–397.

[bb8] Hauf, S., Heisen, B., Aplin, S., Beg, M., Bergemann, M., Bondar, V., Boukhelef, D., Danilevsky, C., Ehsan, W., Essenov, S., Fabbri, R., Flucke, G., Fulla Marsa, D., Göries, D., Giovanetti, G., Hickin, D., Jarosiewicz, T., Kamil, E., Khakhulin, D., Klimovskaia, Kluyver, T., Kirienko, Y., Kuhn, M., Maia, L., Mamchyk, D., Mariani, V., Mekinda, L., Michelat, T., Münnich, A., Padee, A., Parenti, A., Santos, H., Silenzi, A., Teichmann, M., Weger, K., Wiggins, J., Wrona, K., Xu, C., Youngman, C., Zhu, J., Fangohr, H. & Brockhauser, S. (2019). *J. Synchrotron Rad.* **26**, 1448–1461. 10.1107/S1600577519006696 31490132

[bb9] Henkel, A., Maracke, J., Munke, A., Galchenkova, M., Rahmani Mashhour, A., Reinke, P., Domaracky, M., Fleckenstein, H., Hakanpää, J., Meyer, J., Tolstikova, A., Carnis, J., Middendorf, P., Gelisio, L., Yefanov, O., Chapman, H. N. & Oberthür, D. (2022). *Acta Cryst.* A**78**, e560.

[bb10] Kim, E. S. & Yoon, M. (2009). *IEEE Trans. Nucl. Sci.* **56**, 3597–3606.

[bb11] Knoška, J., Adriano, L., Awel, S., Beyerlein, K. R., Yefanov, O., Oberthuer, D., Peña Murillo, G. E., Roth, N., Sarrou, I., Villanueva-Perez, P., Wiedorn, M. O., Wilde, F., Bajt, S., Chapman, H. N. & Heymann, M. (2020). *Nat. Commun.* **11**, 657. 10.1038/s41467-020-14434-6 PMC6994545 32005876

[bb12] Ko, I. S., Kang, H.-S., Heo, H., Kim, C., Kim, G., Min, C.-K., Yang, H., Baek, S. Y., Choi, H.-J., Mun, G., Park, B. R., Suh, Y. J., Shin, D. C., Hu, J., Hong, J., Jung, S., Kim, S.-H., Kim, K., Na, D., Park, S. S., Park, Y. J., Jung, Y. G., Jeong, S. H., Lee, H. G., Lee, S., Lee, S., Oh, B., Suh, H. S., Han, J.-H., Kim, M. H., Jung, N.-S., Kim, Y.-C., Lee, M.-S., Lee, B.-H., Sung, C.-W., Mok, I.-S., Yang, J.-M., Parc, Y. W., Lee, W.-W., Lee, C.-S., Shin, H., Kim, J. H., Kim, Y., Lee, J. H., Park, S.-Y., Kim, J., Park, J., Eom, I., Rah, S., Kim, S., Nam, K. H., Park, J., Park, J., Kim, S., Kwon, S., An, R., Park, S. H., Kim, K. S., Hyun, H., Kim, S. N., Kim, S., Yu, C.-J., Kim, B.-S., Kang, T.-H., Kim, K.-W., Kim, S.-H., Lee, H.-S., Lee, H.-S., Park, K.-H., Koo, T.-Y., Kim, D.-E. & Lee, K. B. (2017). *Appl. Sci.* **7**, 479.

[bb13] Koliyadu, J. C. P., Letrun, R., Kirkwood, H. J., Liu, J., Jiang, M., Emons, M., Bean, R., Bellucci, V., Bielecki, J., Birnsteinova, S., de Wijn, R., Dietze, T., E, J., Grünert, J., Kane, D., Kim, C., Kim, Y., Lederer, M., Manning, B., Mills, G., Morillo, L. L., Reimers, N., Rompotis, D., Round, A., Sikorski, M., Takem, C. M. S., Vagovič, P., Venkatesan, S., Wang, J., Wegner, U., Mancuso, A. P. & Sato, T. (2022). *J. Synchrotron Rad.* **29**, 1273–1283. 10.1107/S1600577522006701 PMC9455201 36073887

[bb14] Kumar, G. & Bhatia, P. K. (2014). *2014 Fourth International Conference on Advanced Computing & Communication Technologies*, pp. 5–12. Piscataway: IEEE.

[bb15] Mancuso, A. P., Aquila, A., Batchelor, L., Bean, R. J., Bielecki, J., Borchers, G., Doerner, K., Giewekemeyer, K., Graceffa, R., Kelsey, O. D., Kim, Y., Kirkwood, H. J., Legrand, A., Letrun, R., Manning, B., Lopez Morillo, L., Messerschmidt, M., Mills, G., Raabe, S., Reimers, N., Round, A., Sato, T., Schulz, J., Signe Takem, C., Sikorski, M., Stern, S., Thute, P., Vagovič, P., Weinhausen, B. & Tschentscher, T. (2019). *J. Synchrotron Rad.* **26**, 660–676. 10.1107/S1600577519003308 PMC6510195 31074429

[bb16] Mancuso, A. P., Aquila, A., Borchers, G., Giewekemeyer, K. & Reimers, N. (2013). Technical Design Report. Scientific Instrument Single Particles, Clusters, and Biomolecules (SPB). European X-ray Free-Electron Laser Facility GmbH, Hamburg, Germany.

[bb17] Mills, G., Bean, R. & Mancuso, A. P. (2020). *Appl. Sci.* **10**, 3642.

[bb18] Milne, C. J., Schietinger, T., Aiba, M., Alarcon, A., Alex, J., Anghel, A., Arsov, V., Beard, C., Beaud, P., Bettoni, S., Bopp, M., Brands, H., Brönnimann, M., Brunnenkant, I., Calvi, M., Citterio, A., Craievich, P., Csatari Divall, M., Dällenbach, M., D’Amico, M., Dax, A., Deng, Y., Dietrich, A., Dinapoli, R., Divall, E., Dordevic, S., Ebner, S., Erny, C., Fitze, H., Flechsig, U., Follath, R., Frei, F., Gärtner, F., Ganter, R., Garvey, T., Geng, Z., Gorgisyan, I., Gough, C., Hauff, A., Hauri, C. P., Hiller, N., Humar, T., Hunziker, S., Ingold, G., Ischebeck, R., Janousch, M., Juranić, P., Jurcevic, M., Kaiser, M., Kalantari, B., Kalt, R., Keil, B., Kittel, C., Knopp, G., Koprek, W., Lemke, H. T., Lippuner, T., Llorente Sancho, D., Löhl, F., Lopez-Cuenca, C., Märki, F., Marcellini, F., Marinkovic, G., Martiel, I., Menzel, R., Mozzanica, A., Nass, K., Orlandi, G. L., Ozkan Loch, C., Panepucci, E., Paraliev, M., Patterson, B., Pedrini, B., Pedrozzi, M., Pollet, P., Pradervand, C., Prat, E., Radi, P., Raguin, J.-Y., Redford, S., Rehanek, J., Réhault, J., Reiche, S., Ringele, M., Rittmann, J., Rivkin, L., Romann, A., Ruat, M., Ruder, C., Sala, L., Schebacher, L., Schilcher, T., Schlott, V., Schmidt, T., Schmitt, B., Shi, X., Stadler, M., Stingelin, L., Sturzenegger, W., Szlachetko, J., Thattil, D., Treyer, D. M., Trisorio, A., Tron, W., Vetter, S., Vicario, C., Voulot, D., Wang, M., Zamofing, T., Zellweger, C., Zennaro, R., Zimoch, E., Abela, R., Patthey, L. & Braun, H.-H. (2017). *Appl. Sci.* **7**, 720.

[bb19] Oberthuer, D., Knoška, J., Wiedorn, M. O., Beyerlein, K. R., Bushnell, D. A., Kovaleva, E. G., Heymann, M., Gumprecht, L., Kirian, R. A., Barty, A., Mariani, V., Tolstikova, A., Adriano, L., Awel, S., Barthelmess, M., Dörner, K., Xavier, P. L., Yefanov, O., James, D. R., Nelson, G., Wang, D., Calvey, G., Chen, Y., Schmidt, A., Szczepek, M., Frielingsdorf, S., Lenz, O., Snell, E., Robinson, P. J., Šarler, B., Belšak, G., Maček, M., Wilde, F., Aquila, A., Boutet, S., Liang, M., Hunter, M. S., Scheerer, P., Lipscomb, J. D., Weierstall, U., Kornberg, R. D., Spence, J. C. H., Pollack, L., Chapman, H. N. & Bajt, S. (2017). *Sci. Rep.* **7**, 44628.

[bb20] O’Gorman, L. (1994). *CVGIP Graph. Models Image Process.* **56**, 494–506.

[bb26] Open CV Team (2023). *OpenCV – Open Computer Vision Library*, https://opencv.org/.

[bb21] Patel, J., Round, A., Bielecki, J., Doerner, K., Kirkwood, H., Letrun, R., Schulz, J., Sikorski, M., Vakili, M., de Wijn, R., Peele, A., Mancuso, A. P. & Abbey, B. (2022). *J. Appl. Cryst.* **55**, 944–952. 10.1107/S1600576722005891 PMC9348884 35974719

[bb22] Pellegrini, C. (2020). *Nat. Rev. Phys.* **2**, 330–331.

[bb23] Rasheed, S., Naeem, A. & Ishaq, O. (2012). *Proceedings of the World Congress on Engineering and Computer Science 2012*, edited by S. I. Ao, C. Douglas, W. S. Grundfest & J. Burgstone, pp. 119–203. Hong Kong: Newswood.

[bb24] Schulz, J., Bielecki, J., Doak, R. B., Dörner, K., Graceffa, R., Shoeman, R. L., Sikorski, M., Thute, P., Westphal, D. & Mancuso, A. P. (2019). *J. Synchrotron Rad.* **26**, 339–345. 10.1107/S1600577519000894 PMC6412181 30855241

[bb25] Spence, J. C. H. (2017). *IUCrJ*, **4**, 322–339. 10.1107/S2052252517005760 PMC5571796 28875020

[bb27] Vakili, M., Bielecki, J., Knoška, J., Otte, F., Han, H., Kloos, M., Schubert, R., Delmas, E., Mills, G., de Wijn, R., Letrun, R., Dold, S., Bean, R., Round, A., Kim, Y., Lima, F. A., Dörner, K., Valerio, J., Heymann, M., Mancuso, A. P. & Schulz, J. (2022). *J. Synchrotron Rad.* **29**, 331–346. 10.1107/S1600577521013370 PMC8900844 35254295

[bb7] Vakili, M. & Otte, F. (2021). *3D Designs for Liquid Sample Delivery at the European XFEL*, https://github.com/flmiot/EuXFEL-designs.

[bb28] Vakili, M., Vasireddi, R., Gwozdz, P. V., Monteiro, D. C. F., Heymann, M., Blick, R. H. & Trebbin, M. (2020). *Rev. Sci. Instrum.* **91**, 085108. 10.1063/5.0012806 32872940

[bb29] Weierstall, U. (2014). *Philos. Trans. R. Soc. B*, **369**, 20130337. 10.1098/rstb.2013.0337 PMC4052872 24914163

