# Supplementary material for: Real-time analysis of liquid jet sample delivery stability for an X-ray free-electron laser using machine vision
Source: J Appl Crystallogr. 2024 Nov 17;57(Pt 6):1859–70. doi: 10.1107/S1600576724009853 (PMC11611288; doi:10.1107/S1600576724009853)
Supplement: Supplementary file 1 [file j-57-01859-sup1.pdf]

JOURNAL OF
APPLIED
CRYSTALLOGRAPHY

Volume 57 (2024)

**Supporting information for article:**

# Real-time analysis of liquid-jet sample-delivery stability for an X-ray free-electron laser using machine vision

**Jaydeep Patel, Adam Round, Raphael de Wijn, Mohammad Vakili, Gabriele Giovanetti, Diogo Filipe Monrroy Vilan e Melo, Juncheng E, Marcin Sikorski, Jayanth Koliyadu, Faisal H. M. Koua, Tokushi Sato, Adrian Mancuso, Andrew Peele and Brian Abbey**

### S1. Integration of machine vision-based jet alignment algorithm into Karabo

Karabo is the facility control system responsible for all the aspects of the EuXFEL instruments and their corresponding components. Karabo incorporates two systems which can access the same peripheral, side-microscope camera whose output is analysed by our algorithm. Here, we were able to work with instrument scientists and engineers to implement our algorithm on the control server of Karabo. This is a precursor step to the algorithm being able to trigger a response from the beamline stepper motors to affect repositioning based on the camera feedback. This Karabo server used for this is referred to as the “middle layer server”.

As with any other automation system this system requires calibration before it can be exploited during a user experiment. Karabo provides graphical user interfaces (GUI) with standardized scenes across the facility for similar devices. Figure S1 shows the screen capture of this Karabo GUI for this algorithm, which was implemented at the SPB/SFX instrument of the EuXFEL.

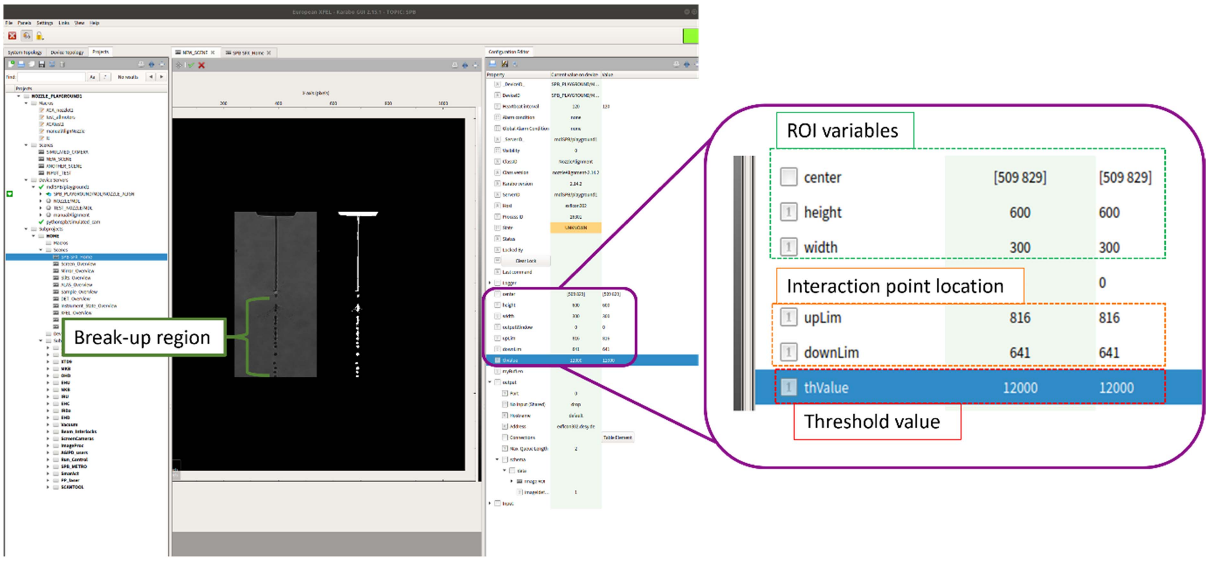


**Figure S1** Screen shot of the GUI in Karabo control system for the jet and beam overlap algorithm, during the initial calibration step of the machine vision algorithm. The GUI enables the user to define the ROI, interaction point and threshold values to optimise the performance of the algorithm for the specific nozzle/jetting/lighting conditions used in the experiment.

During testing of the algorithm to assess its operation in real-time, no measured latency in processing and classifying the images was measured. This indicates that our liquid jet monitoring system could be run in parallel with the standard beamline control systems and data acquisition (DAQ) software without causing any lag to appear in their operation. The lack of latency is the result of significant optimisation of the machine vision algorithm resulting in efficient image processing and feature extraction, providing the ideal approach for real-time assessment of the liquid jet.

**S2. Jet drift with various window sizes.**

In order to achieve the appropriate balance between robustness to frame-to-frame ‘jitter’ and response time, three different moving average window sizes were tested in this study. The most appropriate window size for the data analysed here was found to be a moving average window size of 10 frames (see Fig. S2).

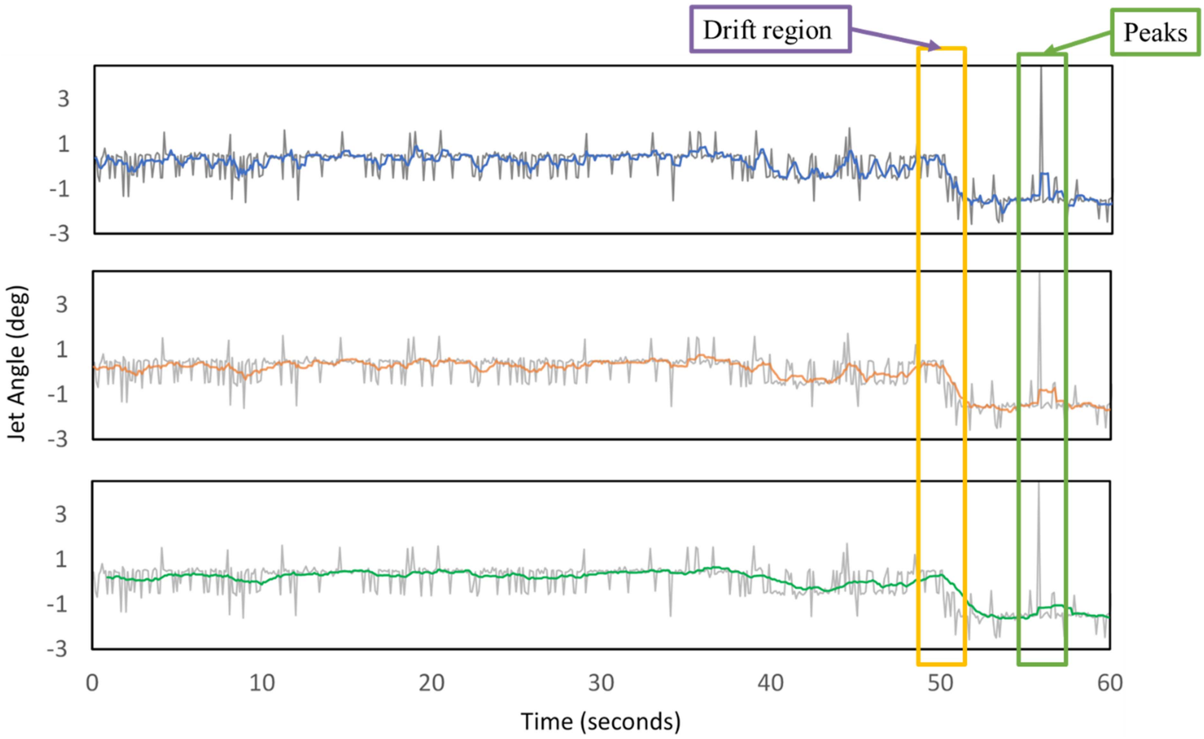


**Figure S2** Graphs showing representative jet angles for a buffer and sample containing 1 µm crystals formed using a DFFN with a 75 µm aperture at a jet speed of 45 m/s. The 3 plots are included to illustrate the effect of increasing the moving average window size. Data shown corresponds to a window size of 5 (top), 10 (middle), and 20 (bottom) frames, equivalent to averaging over 0.5 s, 1 s, and 2 s respectively. The moving average is calculated according to the First In, First Out (FIFO) method.

**Table S1** Table of jetting parameters for all the nozzles used in this experiment.

| Nozzle type | Nozzle Characteristics $D_{liquid}$–$D_{gas}$–$H_{liquid\text{-}gas}$ (µm) | Average Jet Speed (m/s) | He flow rate (mg/min) | Total liquid flow rate (µL/min) *50-50 for DFFN (sample liquid – Ethanol) |
|---|---|---|---|---|
| **GDVN75** | 75–60–75 | 25 | 10 | 80 |
| | | 30 | 13 | 60 |
| | | 40 | 20 | 30 |
| | | 45 | 25 | 30 |
| | | 50 | 32 | 30 |
| **DFFN75** | 75-70-70 | 25 | 13 | 80* |
| | | 30 | 13 | 40* |
| | | 40 | 23 | 25* |
| | | 45 | 39 | 35* |
| | | 50 | 39 | 22* |
| **GDVN100** | 100-75-100 | 25 | 15 | 80 |
| | | 30 | 23 | 80 |
| | | 40 | 39 | 50 |
| | | 45 | 39 | 28 |
| | | 50 | 39 | 17 |

**S3. Simplified schematic of the SPB/SFX beamline and its sample delivery setup.**

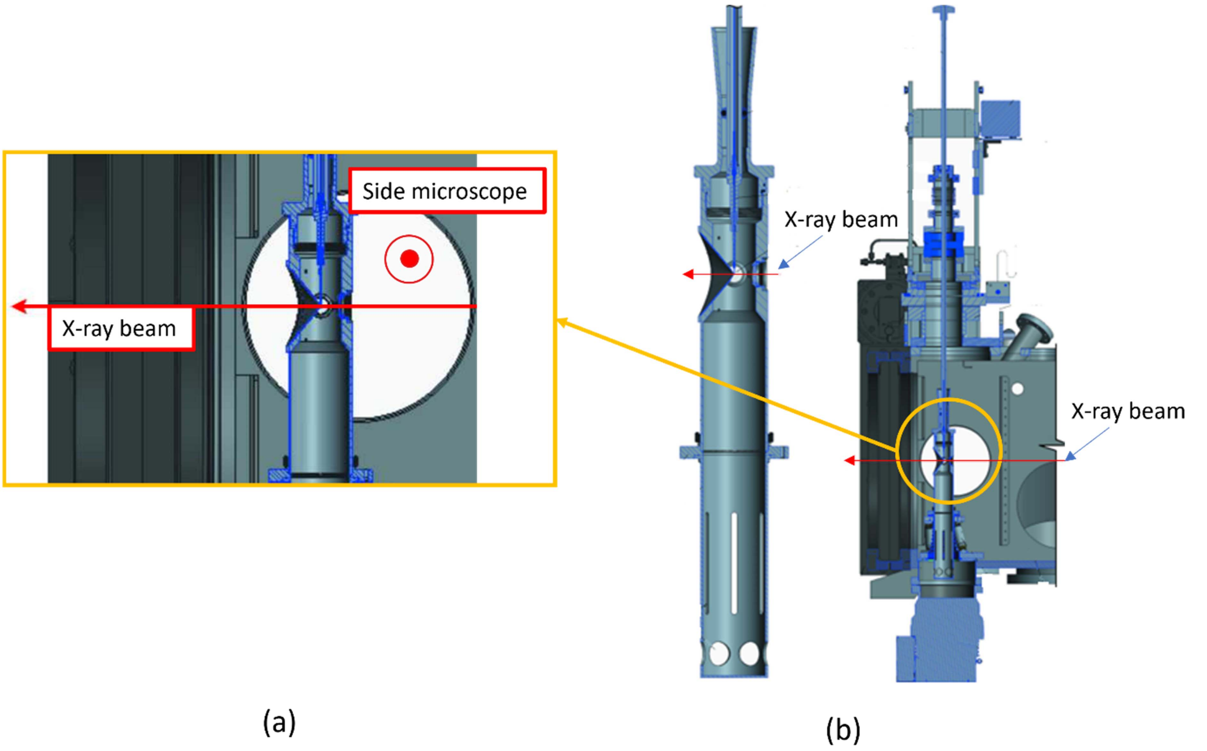


**Figure S3** The image above illustrates the SBP/SFX sample chamber where the X-ray beam and liquid jet interact. This image is acquired and modified from J. Schulz Et al. for simplification. Please refer to this for link further information.

**Supplementary Video S1**. The liquid jet in this video is an example of a “highly unstable jet”, evident from the quick lateral movements of the jet, which is one of the key behavioural traits making a jet completely unusable even with realignment. The best possible action for such cases is to stop jetting and perform a cleaning cycle to remove the clog.

**Supplementary Video S2**. The liquid jet in this video is classified as an "unstable jet" due to its non-continuous nature between the nozzle tip and the interaction region. This classification fits this case because, even though its momentary change in angle is not the issue, the non-continuous nature of the liquid jet between the nozzle tip and the interaction region is. This reduces the overall jet and beam overlap and causes additional sample to be wasted.

**Supplementary Video S3**. The video is a prime example of a "stable jet." It is continuous, does not move away from the interaction region, and the breakup occurs below the interaction region as expected.
